# A four-year trend in pulmonary bacteriologically confirmed tuberculosis case detection in Kampala-Uganda

**DOI:** 10.1186/s12890-019-0853-3

**Published:** 2019-05-10

**Authors:** Nicholas Sebuliba Kirirabwa, Derrick Kimuli, Carol Nanziri, Denis Sama, Syrus Ntudhu, Daniel Ayen Okello, Raymond Byaruhanga, Deus Lukoye, Samuel Kasozi

**Affiliations:** 1 TRACK TB Project, Management Sciences for Health, Plot 15, Princess Anne Drive, Bugolobi, Kampala, Uganda; 2 AIDS Information Centre (AIC), Musajja Alumbwa Road, Mengo, Kisenyi, Kampala, Uganda; 3 Kampala Capital City Authority (KCCA), Public Health and Environment, Plot 1-3 Sir Apollo Kaggwa Road, Kampala, Uganda

## Abstract

**Background:**

The management and control of pulmonary bacteriologically confirmed (PBC) tuberculosis (TB) also known as infectious TB is important not only to monitor for resistance but also to check for severity, treatment response and limit its spread.

**Method:**

A retrospective analysis of diagnosis smear results of PBC TB patients in Kampala district registered between January 2012 and December 2015 at 65 TB diagnosis and treatment units (DTUs) was done.

**Results:**

Of the 10,404 records; 6551 (63.0%) belonged to PBC TB patients, 3734 (57.0%) of whom were male. Sputum smear microscopy was the diagnostic test most commonly used 4905 (74.9%) followed by GeneXpert testing, 1023 (15.6%). Majority, 1951 (39.8%), of the PBC TB patients had a smear positivity grading of 3+ (> 10 acid-fast bacillus (AFB)/Fields). Public facilities diagnosed more PBC TB patients compared to private facilities, 3983 (60.8%) vs 2566 (39.2%). From 2012 through 2015, there was a statistically significant increase in PBC TB patients enrolled on anti-TB treatment from 1389 to 2194 (*p* = 0.000). The percentage of HIV positive co-infected PBC TB patients diagnosed decreased from 597(43%) to 890(40.6%) (*p* = 0.000) within same period. Linkage to HIV care improved from 229 (34.4%) in 2012 to 464 (52.1%) in 2015 (*p* = 0.000). The treatment success rate (TSR) for PBC TB patients improved from 69% in 2012 to 75.5% by end of 2015 (*p* = 0.001) with an improvement in cure rate from 52.3% to 62% (*p* = 0.000). There was an observed significant decrease in TB related mortality from 8.9 to 6.4% (*p* = 0.013).

**Conclusion:**

The proportion of diagnosed PBC TB patients increased from 2012 to 2015. PBC TB patients diagnosed with 3+ smear positivity grading results consistently contributed to the highest proportion of diagnosed PBC TB patients from 2012 to 2015. This could be due to the delay in diagnosis of TB patients because of late presentation of patients to clinics. A prospective study of PBC TB patients diagnosed with 3+ smear positivity grading may elucidate the reasons for the delay to diagnosis. Further, we propose a study of wider scope to estimate how many people a single PBC TB patient is likely to infect with TB before being diagnosed and treated.

## Background

Tuberculosis (TB) remains a global public health problem despite the presence of TB pharmacotherapy for more than 50 years and the use of vaccines for more than 90 years [1, 2]. According to the World Health Organization (WHO), TB remains one of the major global health threats of the twenty-first century [3] as well as a major cause of socio-economic distress [4, 5]. According to the 2016 Global Tuberculosis (TB) Report, in 2016 alone an estimated 10.4 million people developed TB and 1.4 million died from the disease with over 95% of these deaths occurring in low- and middle-income countries. An estimated 11% of the incident TB cases in 2015 were concurrently human immunodeficiency virus (HIV) positive [6].

TB has remained a public health challenge in low resource settings and, Uganda, ranked as one of the highest TB and TB/HIV burden countries in the world, remains disproportionately affected [6]. Uganda's TB prevalence is estimated at 253 per 100, 000 population [6]. Since 1998, the Uganda National Tuberculosis and Leprosy Program (NTLP) has implemented a series of WHO TB control strategies. The first of which was the Directly Observed Treatment Short Course (DOTS) strategy, which allows patients to take their daily drugs under the observation of health professionals, thereby improving treatment compliance and increasing the cure rate [7, 8]. This was replaced by the more comprehensive Stop TB Strategy implemented in 2006, followed by the End TB Strategy in 2015, to address the emerging challenges of drug-resistant TB, TB/HIV co-infection and the incorporation of new diagnostic tests, such as GeneXpert [9].

Reports have shown that PCB TB patients are more likely to transmit TB to susceptible hosts compared to those with a bacteriologically negative status [10]. A high proportion of PBC TB cases might imply more reliance on bacteriological examination to diagnose TB or a delay in diagnosis of both extrapulmonary (EP) and clinical pulmonary (PCD) TB which with time become easier to diagnose bacteriologically. On the other hand, low proportions may reflect several gaps in diagnosis such as lack of capacity by the program to accurately diagnose TB through bacteriological examination. Such programs majorly rely on clinical diagnosis basing on the presence of radiographic abnormalities on the radiograph or based on a high index of clinical suspicion which carries along with risks of falsely treating patients with other conditions for tuberculosis [11].

Although the proportion contributed by PBC TB patients to the total case notification is important in the monitoring of the quality of the national program, the trend of their notification has not been carefully studied to further inform the epidemiology of TB in this setting. We sought to determine the trend and outcomes of PBC TB over the four-year period of January 2012 –December 2015 in Kampala City.

## Methods

### Study design

This retrospective analysis examined TB patient-records in Kampala district for the calendar years January 2012 to December 2015. This audit was conducted at all of the six TB control divisions in Kampala district (Mulago, Central, Nakawa, Kawempe, Lubaga, and Makindye).

### Study site

Data were collected from 65 TB diagnosis and treatment units (DTUs) in the six TB control divisions of Kampala district.

Kampala has a total population of approximately 1.5 million inhabitants by night; estimated to double during the day due to large numbers of people that come to work, conduct various business, or seek various services. Kampala district is surrounded by Wakiso district. A good proportion of TB patients come to seek medical services from Wakiso to Kampala [12]. National data shows that the burden of TB in Kampala is disproportionately high compared to the other districts in the country [13]. While the population of Kampala accounts for 4.3% of the national population, 17% of the total number of TB cases that are reported to the Ministry of Health derive from Kampala [13].

#### Study population

All TB patients registered between January 2012 and December 2015 among the 65 DTUs in Kampala were included and their bacteriological status was collected.

#### Data variables, collection, and source of data

Information on smear results and demographics such as age, sex, and place of residence were collected from the Unit TB patients register. Data on patient bacteriological status (PBC, PCD or EP) and HIV status were abstracted and matched from both the health unit and laboratory TB registers. For this study, a smear diagnosis of 1+ represents 9 Acid Fast Bacilli (AFB)/100 fields (scanty), 2+ represents 10–99 AFB/Fields (moderate) and 3+ represents > 10 AFB/Fields (numerous). For the purpose of the study, TB/HIV patients that were documented to have received Co-trimoxazole preventive therapy (CPT) or Antiretroviral therapy (ART) were considered as linked to HIV care. Treatment outcomes (cured, completed, died, lost to follow up, not evaluated and failure) of patients were also abstracted. Patients with a cure or completed outcome were categorized to have completed treatment successfully (treatment success rate) while other categories were classified as unfavorable patient outcomes.

### Data entry and statistical analysis

A two-person independent data cleaning and verification was conducted in the health facilities. Errors and omissions in the data were corrected using primary data source documents at the DTUs. The data were coded and entered by trained data entry clerks under the supervision of the investigators into a pre-prepared computer Excel document. The Excel document was merged and exported to a Statistical Package for the Social Sciences (SPSS) version 16 software for subsequent analysis. Frequencies were tabulated to determine the trends of PBC TB by six TB control divisions, sex and age group. Mantel–Haenszel Chi Statistics and Odds ratios were calculated at 95% confidence intervals to test relevant relationships. The national average PBC TB notification rate over the four-year period was used to classify divisions as having a PBC TB notification rate of either above or below the national average PBC TB notification rate.

## Results

We assessed a total of 10,404 records of TB patients from the 65 active DTUs in the five divisions of Kampala during the period of January 2012 to December 2015. Of the 10,404 records of TB patients; 6551 (63.0%) were PBC, 2207 (21.2%) were pulmonary clinically diagnosed (PCD) and 1343 (12.9%) were extrapulmonary (EP) while 2.9% had no documentated classification of TB in the register.

The PBC TB patients had a mean age of 31.92 (SD ±11.24) years. The majority of PBC TB patients were male, 3734(57.0%). PBC TB patients contribution by division from highest to lowest was Kawempe, Lubaga, Makindye, Nakawa, and Central respectively, (see Table 1 for details). About 74.9% of all PBC TB patients were diagnosed by sputum smear microscopy test, 15.6% were diagnosed by GeneXpert testing, and only four patients (0.1%) were diagnosed by culture testing. The remaining records were undocumented and classified as unknown.

**Table 1:** Characteristics of all pulmonary bacteriologically confirmed TB patients who registered between January 2012 and December 2015

Background characteristics	*n* (%)
Total, *n*	6551
Residence of TB patients by division	
Kawempe	1807(27.6)
Lubaga	1206(18.4)
Makindye	1140(17.4)
Nakawa	649(9.9)
Central	401(6.1)
Others	1348(20.6)
Sex	
Female	2817(43.0)
Male	3734(57.0)
Age (years)	
< 5	20(0.3)
5–19	605(9.2)
20–34	3572(54.5)
35–49	1872(28.6)
50–64	358(5.5)
> 65	96(1.5)
Missing	28(0.4)
Mean Age	31.92 ± SD 11.24
HIV serostatus	
Positive	2843(43.4)
Negative	3593(54,6)
Unknown	115(1.76)
Diagnostic test	
Sputum smear microscopy	4905(74.9)
GeneXpert test	1023(15.6)
Culture test	4(0.1)
Unknown	619(9.4)
Type of facility	
Private	2566(39.2)
Public	3985(60.8)

Source: Primary Data. *TB-*Tuberculosis*, HIV*-Human Immunodeficiency Virus

### Trends of TB patient enrollment by disease classification

Throughout the period of January 2012 to the end of 2015, the number of PBC TB patients enrolled for treatment per year increased from 1389 (58.4%) to 2194 (66.8%) (*p* = 0.000). The number of PCD TB patients enrolled on anti-TB treatment was also observed to have attained a statistically significant increase per year from 418 (17.6%) to 745 (22.7%) patients between the years under review. While the number of EP TB patients and those with unknown disease classification had a statistically significant decrease per year from 437 (18.4%) and 136 (5.7%) to 342 (10.4%) and 5 (0.2%) respectively, (Table 2).

**Table 2:** Patient started on anti-TB treatment by TB diagnostic class

Characteristic	2012 *n* (%)	2013 *n* (%)	2014 *n* (%)	2015 *n* (%)	*P* value
PBC	1389 (58.4)	1244 (55.8)	1724 (68.7)	2194 (66.8)	0.000^a^
PCD	418 (17.6)	568 (25.5)	476 (19.0)	745 (22.7)	0.005^a^
EP	437 (18.4)	319 (14.3)	245 (9.8)	342 (10.4)	0.000^a^
Unknown	136 (5.7)	97 (4.4)	65 (2.6)	5 (0.2)	0.000^a^

Source: Primary Data, 95% Confidence Interval. *TB*-Tuberculosis, *PBC*-Pulmonary Bacteriologically Confirmed Tuberculosis, *PCD*-Pulmonary Clinically Diagnosed Tuberculosis, *EP*-Extrapulmonary Tuberculosis

^a^Statistically Significant Variable

### Diagnostic tests done for PBC cases

Diagnosis methods included; microscopy, GeneXpert, and culture. There was a decreasing trend in the use of microscopy and an increasing trend in the use of GeneXpert between 2012 and 2015. In 2012, all PBC TB patients diagnosed by microscopy were 1289 (92.8%) vs 1377 (62.8%) in 2015. The proportion of PBC patients diagnosed with GeneXpert increased from 66 (5.3%) in 2013 to 610 (27.8%) in 2015 (Table 3).

**Table 3:** Number (proportion) of diagnostic tests done for pulmonary bacteriologically confirmed TB cases

Test	2012 *n* (%)	2013 *n* (%)	2014 *n* (%)	2015 *n* (%)	*P* value
Smear Microscopy	1289 (92.8)	1063 (85.5)	1176 (68.2)	1377 (62.8)	< 0.000^a^
GeneXpert	1 (0.1)	66 (5.3)	346 (20.1)	610 (27.8)	< 0.000^a^
Culture	0 (0.0)	0 (0.0)	0 (0.0)	4 (0.2)	0.050
Unknown	99 (7.1)	115 (9.2)	202 (11.7)	203 (9.3)	0.000^a^

Source: Primary Data, 95% Confidence Interval

^a^Statistically Significant Variable

### Sputum smear positivity grading

There was a percentage decline in PBC TB patients diagnosed with sputum smear positivity grade results of 3+ (> 10 AFB/Fields) and 2+ (10–99 AFB/Fields) per year from 42.1% in 2012 to 38.3% in 2015 (*p* = 0.215) and 31.7% in 2012 to 30.4% in 2015 (*p* = 0.291) respectively. Those with sputum smear positivity grade results of 1+ (9 AFB/100 fields) had a significant increase from 26.1% in 2012 to 31.3% in 2015 (*p* = 0.003), (See details in Table 4).

**Table 4:** Results of microscopy sputum examination grading by number of patients

Test	2012 *n* (%)	2013 *n* (%)	2014 *n* (%)	2015 *n* (%)	*P* value
3+	543 (42.1)	420 (39.5)	461 (39.2)	527 (38.3)	0.215
2+	409 (31.7)	310 (29.2)	334 (28.4)	419 (30.4)	0.291
1+	337 (26.1)	333 (31.3)	381 (32.4)	431 (31.3)	0.003^a^

Source: Primary Data, 95% Confidence Interval

^a^Statistically Significant Variable

### Diagnosis of PBC TB by facility type

Findings showed more PBC TB patients diagnosed from public facilities compared to private facilities (See Table 1). Throughout the years 2012 to 2015, the percentage of PBC TB patients diagnosed from public facilities increased from 790 (56.9%) to 1465 (66.8%), (*p* = 0.000). Meanwhile, the percentage of PBC TB patients diagnosed from private facilities decreased from 599 (43.1%) to 729 (33.2%), (*p* = 0.000), (See details in Table 5).

**Table 5:** Percentage of pulmonary bacteriologically confirmed TB patients by diagnostic facility

Test	2012 *n* (%)	2013 *n* (%)	2014 *n* (%)	2015 *n* (%)	*P* value
Private	599 (43.1)	525 (42.2)	713 (41.4)	729 (33.2)	0.000^a^
Public	790 (56.9)	719 (57.8)	1011 (58.6)	1465 (66.8)	

Source: Primary Data, 95% Confidence Interval

^a^Statistically Significant Variable

### HIV status for PBC TB patients and linkage to HIV care services

Although there was an increase in absolute numbers of PBC TB patients that tested HIV positive, a decline in the proportion that tested HIV positive was observed from 597 (43%) to 890 (40.6%) between 2012 and 2015 respectively (*p* = 0.000). The number of PBC TB/HIV co-infected patients linked to care improved from 229 (34.4%) in 2012 to 464 (52.1%) in 2015 (*p* = 0.000). The proportion of PBC TB/HIV co-infected patients on CPT improved from 225 (37.7%) in 2012 to 462 (51.9%) in 2015 (*p* = 0.000). Likewise, TB/HIV patients on ART improved from 212 (35.5%) to 431 (48.4%) for 2012–2015, See Table 6 for details.

**Table 6:** Patients’ HIV status and linkage to HIV care

Characteristic	2012 *n* (%)	2013 *n* (%)	2014 *n* (%)	2015 *n* (%)	*P* value
HIV Serostatus (n)	1389	1244	1724	2194	
Positive	597 (43.0)	608 (48.9)	782 (45.4)	890 (40.6)	0.000^a^
Negative	739 (53.2)	620 (49.8)	935 (54.2)	1299 (59.2)	0.000^a^
Unknown	53 (3.8)	16 (1.3)	7 (0.4)	5 (0.2)	0.000^a^
Linked to care	597	608	782	890	
Yes	229 (34.4)	320 (52.6)	381 (48.7)	464 (52.1)	0.000^a^
On CPT					
Yes	225 (37.7)	315 (51.8)	378 (48.3)	462 (51.9)	0.000^a^
On ART					
Yes	212 (35.5)	303 (49.8)	364 (46.5)	431 (48.4)	0.000^a^

Source: Primary Data, 95% Confidence Interval. HIV-Human Immunodeficiency Virus, CPT-Co-trimoxazole Preventive Therapy, ART-Antiretroviral Therapy

^a^Statistically Significant Variable

### Treatment outcomes

The treatment success rate (TSR) for PBC cases increased from 69% in 2012 to 75.5% by the end of 2015 (*p* = 0.001). The cure rate also improved from 52.3 to 62% (*p* = 0.000) during the same period. The percentage of PBC TB patients not evaluated by the time of establishing patient outcomes decreased from 12.4% in 2012 to 9.7% (*p* = 0.069) in 2015. Likewise, the number of PBC TB patients who died during treatment had a steady decrease from 8.9 to 6.4% (*p* = 0.013), see details in Table 7.

**Table 7:** Treatment outcomes for pulmonary bacteriologically confirmed TB patients from 2012 to 2015

Outcome	2012 *n* (%)	2013 *n* (%)	2014 *n* (%)	2015 *n* (%)	*p*-value
Total	1389	1244	1724	2194	
Cured	726 (52.3)	743 (59.7)	985 (57.1)	1360 (62.0)	0.000^a^
Completed Treatment	233 (16.8)	185 (14.9)	233 (13.5)	296 (13.5)	0.004^a^
TSR	959 (69.0)	928 (74.6)	1218 (70.6)	1656 (75.5)	0.001^a^
Unfavorable	430 (31.0)	316 (25.4)	506 (29.4)	538 (24.5)	0.001^a^
Died	123 (8.9)	80 (6.4)	120 (7.0)	140 (6.4)	0.013^a^
Failure	31 (2.2)	20 (1.6)	36 (2.1)	40 (1.8)	0.568
Lost to follow up	106 (7.6)	83 (6.7)	126 (7.3)	145 (6.6)	0.363
Not evaluated	170 (12.4)	133 (10.7)	224 (13.0)	213 (9.7)	0.069

Source: Primary Data, 95% Confidence Interval. TSR – Treatment Success Rate

^a^Statistically Significant Variable

## Discussion

This study analyzed TB patient records from January 2012 to December 2015 in all registered DTUs of the most highly burden district in Uganda. Our discussion of PBC TB patients gives detail that according to our knowledge has not been examined before in this country and district. As widely observed throughout the world and in the country, most of the PBC TB patients were male [14–16] in their young and productive years [15]. This is emphasized by the clear-cut gender differential that has been observed in many other studies [14, 16, 17] and the economic impact of TB due to its high incidence among people in the productive years [17].

Majority of the PBC patients were from Kawempe division, this could be due to the location of Mulago National Referral Hospital, the only national referral hopsital in the country, which also contributes to the highest number of TB cases notified each year in the district. About 2% of the PBC TB patients had an unknown/undocumented HIV status and it was noted that less than half of all PBC TB patients were HIV positive patients, a finding that was not surprising since it has been earlier noted by researchers that HIV positive TB patients are most likely to show up with EP TB [18].

The statistically significant increase in PBC TB patients diagnosed across the period for this study could be due to improvement in the quality of microscopy done leading to a better performance in the diagnosis of TB using smear microscopy. This could further be attributed to the NTLP’s adoption of the use of GeneXpert machines to diagnose TB where 15.6% of all PBC patients were diagnosed by GeneXpert. The program and its partners have installed over 13 GeneXpert machines within the district and released new guidelines in 2017 for the adoption of GeneXpert as the first diagnosis method for TB [19]. Given that GeneXpert has a higher sensitivity than microscopy [20], this could account for the registered increase in PBC TB pattients. It is to the knowledge of the authors of this paper that the NTLP moved forward to classify all patients that were GeneXpert positive as PBC patients [21] despite the fact that GeneXpert machines were in use before this decision was made. This may have affected the number of patients classified as PBC for a short period. It is worth noting that there was a significant increase in all classifications of TB within the study period.

We observed that through the study period, patients diagnosed with a 3+ (>10AFB) were persistently higher compared to those that were 2+ or 1+. The authors speculate that this could be caused by late diagnosis due to patient delay in seeking care, leading to an increase in the severity of infection, thus leading to more bacilli detection per length through microscopy. This possibility of total delay in seeking health care and diagnosis may contribute to unfavorable treatment outcomes [22]. In addition, a late diagnosis has implications on cross-transmission of TB to the rest of the population, health workers and other patients at the health centers [23]. However, the same results when expressed as proportions for each year revealed an increase in patients being diagnosed 1+ compared to 2+ and 3+ by the year 2013 that may be attributed to the initiation of active case finding/TB screening using community linkage facilitators (CLFs) leading to early case detection. The contribution of active case finding/TB screening may further be realized by the general decline in proportions of 3+ PBC TB cases diagnosed through the years. This may also be explained by the fact that there were less severely sick patients reporting to the facilities making it less likely diagnose patients as 3+ PBC TB compared to the baseline year 2012 where more severely sick patients would easily be diagnosed. Therefore, this observation could be attributed to both the use of active case finding and use of CLFs.

In Kampala, engagement of the private health provider is important in TB control. Most patients are commonly known to first seek care through private health care providers. There was a decline in PBC TB diagnoses by private facilities in 2015 that this study did not explore further. Researchers, however, speculate that this could be due to the commencement of referral of specimen from private facilities to public facilities for GeneXpert testing. Our research showed a statistically significant increase in GeneXpert testing through the years and this could have influenced the increasing trend of PBC TB patients observed during our study period. This also proved that smear microscopy alone could lead to underestimation of the burden of smear-positive pulmonary TB. More sensitive testing methods such as GeneXpert could provide a better estimate.

Over the years, there was a statistically significant increase in the number of TB/HIV co-infected PBC patients that were linked to HIV care. The proportion of PBC TB/HIV co-infected patients on CPT and ART improved from 37.7% to 51.9% and from 35.5% to 48.4% respectively. This observation could be accounted for by the emphasis on TB/HIV collaborative services by the NTLP and its partners. However, these results still reflected a low uptake of both CPT and ART. This may also be attributed to poor record keeping since many of the PBC TB/HIV co-infected patients did not have a documented status of their CPT and ART uptake.

There was an overall statistically significant improvement in cure rates and TSR throughout the study. This may be attributed to the URBAN DOTS approach that was implemented in Kampala starting 2012. There was an overall decline in death rate of PBC TB patients that was below the 10% that is reported for all TB cases. This probably meant better chances of survival for PBC TB patients compared to all clases of TB patients combined. Public DTUs reported an overall better improvement in TSR compared to private facilities even though they handled more PBC patients thus highlighting the need to bridge the public private partnership gap in TB management.

The major limitation of our study was that over 26% of all the patients that were documented to be PBCs had unknown/undocumented diagnostic test type. This improper documentation may have misclassified patients to PBC that would have otherwise been classified as clinically diagnosed. The severity of PBC patients diagnosed by GeneXpert was not explored because it was documented by most facilities either Positive or Negative only, therefore, our study could not examine this trend, yet it would have clarified a more representative trend in the severity of PBC TB among patients diagnosed by GeneXpert.

### Implication of findings

PBC TB patients are a reservoir for aerosol transmission of MTB infection and a primary emphasis on TB infection control in communities and crowded spaces. Late diagnosis of PBC TB is a likely influence on increased TB spread and the overall prevalence of TB in a population. Until this assessment, there had not been any effort made to document trends of positivity grading of PBC TB patients diagnosed in Uganda and very little of this kind of information has been shared from other findings elsewhere [24]. The NTLP’s adoption of GeneXpert testing as the primary method of TB diagnosis for all presumptive TB patients will probably make the challenges of documentation of degree of smear positivity of PBC TB at a diagnosis more difficult due to the lack of emphasis made on the classification of GeneXpert positive results in some of the established recording tools. This may call for a revision of data collection tools and emphasis on proper recording for laboratory workers, TB clinic workers, and division supervisors. Establishment of the severity of PBC TB at diagnosis or treatment may also help guide the priority for contact tracing in limited resource settings.

## Conclusions

The study sought to analyze and profile PBC TB trends in Kampala over the study period. The proportion of PBC patients increased throughout the years under review. PBC TB patients diagnosed with smear microscopy positivity grading as 3+ consistently contributed to a higher population of those diagnosed as PBC throughout the years compared. This called to attention the consistency in the delayed seeking of care among PBC TB patients and is a stumbling block for effective TB control. We recommend a study of wider scope to estimate how many people a single PBC TB patient is likely to infect with TB per year before s/he is diagnosed and treated. This is crucial in estimating risk and developing strict policies on early detection of PBC TB in this population. GeneXpert testing was a possible contributor to the increase in PBC cases and a challenge to the relevance of microscopy in TB diagnosis [25]. Albeit, we recommend adoption/emphasis of better results reporting options such as MTB detected low, MTB detected intermediate or MTB detected high into the national reporting tools and guidelines used by health workers. This will help capture the severity of the disease at diagnosis in the patient as well as be a marker to late diagnosis/seeking of care.

## Abbreviations

AFB: Acid-Fast Bacillus
ART: Anti-Retroviral Therapy
CPT: Cotrimoxazole Preventive Therapy
DOTS: Directly Observed Treatment Short Course
DTU: Diagnostic and Treatment Unit
EPTB: Extrapulmonary Tuberculosis
HIV: Human Immune-deficiency Virus
MDG: Millennium Development Goal
MTB: *Mycobacterium tuberculosis*
MoH: Ministry of Health
NTLP: National Tuberculosis and Leprosy Program
PBC: Pulmonary Bacteriologically Confirmed Tuberculosis
PCD: Pulmonary Clinically Diagnosed Tuberculosis
TB: Tuberculosis
TSR: Treatment success rate
WHO: World Health Organisation
